# CoAl_2_O_4_/Kaoline Hybrid Pigment Prepared via Solid-Phase Method for Anticorrosion Application

**DOI:** 10.3389/fchem.2018.00586

**Published:** 2018-11-29

**Authors:** Anjie Zhang, Bin Mu, Xiaowen Wang, Aiqin Wang

**Affiliations:** ^1^Key Laboratory of Clay Mineral Applied Research of Gansu Province, Center of Eco-material and Green Chemistry, Lanzhou Institute of Chemical Physics, Chinese Academy of Sciences, Lanzhou, China; ^2^Center of Materials Science and Optoelectronics Engineering, University of Chinese Academy of Sciences, Beijing, China

**Keywords:** CoAl_2_O_4_, hybrid pigments, kaoline, anticorrosion coating, acetic acid-salt fog

## Abstract

In this study, kaoline is incorporated to prepare CoAl_2_O_4_/kaoline hybrid pigments *via* traditional solid-state reaction, and the introduction of kaoline decreases the preparation temperature for formation of spinel CoAl_2_O_4_, and reduces the production cost of cobalt blue as well. More importantly, kaoline may participate in the crystallization of spinel CoAl_2_O_4_ during calcining process, and the hybrid pigments prepared using 8.1% Co_3_O_4_ and 81.5% kaoline features bright blue and good chemical resistance. Due to the synergistic effect between the sheet-like kaoline and the loaded CoAl_2_O_4_, the as-prepared CoAl_2_O_4_/kaoline hybrid pigments can be incorporated into epoxy paint system to obtain the high-performance blue anticorrosion coating, especially for acetic acid-salt fog corrosion.

## Introduction

It is well-known that corrosion of the metal materials is one of the common and key problems to industry applications, especially the high risk fields including aviation, ocean, and chemical industry, etc. (De Leon et al., 2012; Daham et al., 2014; Hao et al., 2017). The organic anticorrosion coating has been recognized as the most effective and economical method for metal protection (Vesely et al., 2010; Golru et al., 2015; Gu et al., 2015; Madhup et al., 2017). The anticorrosion mechanisms of the coatings usually include protection of barrier type, inhibition type protection and electrochemical protection (Al-Sabagh et al., 2017), and the organic anticorrosion coating is served as a physical barrier between corrosive electrolyte and steel substrate. However, the main disadvantage of the organic anticorrosion coating, especially epoxy system, is the creation of holes and defects over the film due to the high crosslink density.

Several approaches have been proposed to enhance the anticorrosion properties of organic anticorrosion coating. By contrast, incorporation of inorganic pigments is efficient and economical way (Kartsonakis et al., 2012; Montemor et al., 2012; Jeon et al., 2013; Naderi et al., 2014). It is conducive to improving the aesthetics (e.g., gloss, opacity, and color) and the mechanical properties of the film (El Saeed et al., 2012). However, the relevant application of some commercial available inorganic pigments is limited due to their disadvantages, such as poor adhesive force and optical transparency, weak abrasion and scratch resistance performance (Zhou et al., 2002; Cayton and Sawitowski, 2005; El-Wahab et al., 2009). Therefore, many attempts have been carried out to prepare high-performance pigments with good anticorrosion properties by designing lamellar-based nano-materials preventing water, oxygen, or ions from penetrating film (Kalendová et al., 2008; Ahmed et al., 2012; Ammar et al., 2016; Zhang et al., 2016).

Cobalt aluminate (cobalt blue, CoAl_2_O_4_) is a spinel type structure blue eco-friendly pigments with excellent thermal and chemical stability, and it has been widely used in the fields of ceramics, plastics, paint, rubber and glass (Jafari and Hassanzadeh-Tabriz, 2014; Álvarez-Docio et al., 2017; He et al., 2017; Yoneda et al., 2018). Therefore, cobalt blue may be expected to develop the high-performance anticorrosion coating, especially for acetic acid-salt fog corrosion. However, the agglomeration of CoAl_2_O_4_ nanoparticles is inevitable during preparation process, which goes against the dispersion of cobalt blue in coating substrate. In addition, the high-cost of cobalt blue also limits its wide application in anticorrosion coating (Tirsoaga et al., 2011; Dandapat and De, 2012; Zou and Zheng, 2016). In our previous work (Mu et al., 2015; Zhang A. J. et al., 2017), clay minerals were employed to fabricate the high-performance CoAl_2_O_4_ hybrid pigment with low-cost and prefect color properties. Kaoline (Kaol) is a 1:1 type layered silicate mineral with one tetrahedral sheet of silica (SiO_4_) linked through oxygen atoms to one octahedral sheet of alumina (AlO_6_) octahedral (Liu et al., 2016; Kloprogge, 2017; Zhang S. L. et al., 2017). Owing to the advantages of excellent mechanical properties, thermal stability, high whiteness and unique flake-like morphology (Vesely et al., 2010; Ahmed et al., 2012; Qu et al., 2017), it is a promising candidate for preparation of cobalt blue hybrid pigment to design the color high-performance anticorrosion coating (Zhang et al., 2018a,b). However, the preparation process of co-precipitation usually involves in the discharge of the wastewater and lengthy steps including washing, solid-liquid separation and drying.

In this study, CoAl_2_O_4_/Kaol hybrid pigments were prepared by the traditional solid-state reaction after grinding the mixture of γ-Al_2_O_3_, Co_3_O_4_, and Kaol. The preparation conditions were systematically investigated including the grinding time, the calcining temperature and the added amount of Kaol. Furthermore, the formation and coloring mechanisms of CoAl_2_O_4_/Kaol hybrid pigments were also studied and discussed. In order to evaluate the anticorrosion application of the as-prepared hybrid pigment, it was incorporated into the epoxy paint system to obtain the blue anticorrosion coating, and then placed in acetic acid-salt spray testing chamber.

## Materials and methods

### Materials

Kaol was obtained from Longyan kaoline Development Co., Ltd., (FuJian, China), Kaol were firstly crushed and purified by 4% HCl (wt%), and then the solid were filtered by passing through a 200-mesh sieve to remove quartz sand, and the XRF (X-ray fluorescence) chemical compositions of Kaol were presented in Table S1 (see ESI) before and after being treated by HCl. γ-Al_2_O_3_ (purity > 99.9%, particle size = 20 ~ 50 nm) and Co_3_O_4_ (purity > 99.9%, D50: 4 ~ 6 μm) were obtained from Shanghai Reagent Factory (Shanghai, China).

### Preparation of CoAl_2_O_4_/Kaol hybrid pigments

8.00 g of Kaol, 0.80 g of Co_3_O_4_ and 1.02 g of Al_2_O_3_ with a Co to Al mole ratio of 2:1 were mixed and grinded in a mortar mill (CRINOER, MG100, China) for 2 h in anhydrous ethanol medium, and then the mixture was calcined to obtain CoAl_2_O_4_/Kaol hybrid pigments. The optimum preparation conditions were systematically investigated including the calcining temperatures (900, 1,000, 1,100, and 1,200°C), the grinding time (0.5, 1, 2, and 4 h) and the added amount of Kaol (0, 1, 2, 4, 6, and 8 g), as summarized in Table 1.

**Table 1:** Conditions for preparation of the samples.

**Factors**	**Temperature/°C**	**Co_3_O_4_/g**	**Al_2_O_3_/g**	**Kaol/g**	**Grinding time (h)**
Temperature	900	0.8	1.02	1	2
	1,000	0.8	1.02	1	2
	1,100	0.8	1.02	1	2
	1,200	0.8	1.02	1	2
Added amount of Kaol	1,100	0.8	1.02	0	2
	1,100	0.8	1.02	1	2
	1,100	0.8	1.02	2	2
	1,100	0.8	1.02	4	2
	1,100	0.8	1.02	6	2
	1,100	0.8	1.02	8	2
Grinding time	1,100	0.8	1.02	1	0.5
	1,100	0.8	1.02	1	1
	1,100	0.8	1.02	1	2
	1,100	0.8	1.02	1	4

### Characterization

The Fourier Transform infrared (FTIR) spectra were collected on a Thermo Nicolet NEXUS TM spectro photometer using KBr pellets. The morphology was observed using transmission electron microscopy (TEM, JEM-1200EX/S, JEOL). XRD test was conducted on X'pert PRO diffractometer with a scan step size of 0.02° per second. Raman spectra were tested using a Labram HR Evolution Raman spectrometer (Horiba). The chemical compositions were measured on a MiniPal 4 XRF spectrometer (PANalytical Co., Netherland). The anticorrosion performance was evaluated on a salt spray testing chamber (YWX-750, Nanjing Huanke experimental equipment Co. Ltd, China). The colorimetric values and reflectance spectra were measured on a Color-Eye automatic differential colorimeter (X-Rite, Ci 7800).

### Stability evaluation of CoAl_2_O_4_/Kaol hybrid pigments

In order to evaluate the environmental stability of CoAl_2_O_4_/Kaol hybrid pigment, it was sprayed onto glass substrate and dried in the open atmosphere after being ultrasonically dispersed into ethanol for 30 min. And then the glass plates were placed for 15 days in a UV Accelerated Weathering Tester (ZN-P, Xinlang, Shanghai, China) with eight UV-B (280-315 nm) bulbs (40 W) at 60°C under UV-B exposure with a radiation intensity of 320 W/m^2^. The color properties were measured before and after being exposed in a UV accelerated weathering tester to evaluate UV irradiation stability.

The obtained sample plates were also immersed into 3M HCl, 3M NaOH, and ethanol at room temperature for 72 h to study the chemical stability of CoAl_2_O_4_/Kaol hybrid pigment, respectively. Unlike with above glass substrate, the hybrid pigments were sprayed onto ceramic substrate and then placed in a muffle furnace to be calcined at 1,000°C for 2 h to evaluate the thermal stability.

### Anticorrosion evaluation of the blue epoxy coating

The anticorrosion coating was prepared by following procedures: Firstly, epoxy resin, 5 wt% CoAl_2_O_4_/Kaol hybrid pigment, dispersing agent, flatting agent, auxiliary solvents, talcum powder, ethyl acetate, and some grading beads added in a 500 mL stainless steel under high shear for 30 min (2,000 rpm), and then the antifoaming agent, wetting agent were added into the above mill base and stirred under low shear for 60 min (500 rpm). If the maximum pigment size was lower than 30 μm, the grinding was considered for meet the requirements, and then the viscosity of the coating was adjusted by ethyl acetate to 20~30 s. And then it was sprayed onto steel plate substrate of size 15 cm × 10 cm × 0.1 cm and dried under the standard conditions (temperature: 25°C and humidity: 40%) for 7 days (the coating thickness: D = 50 ± 2 μm). The obtained sample plates were placed in salt spray testing chamber to evaluate the salt fog corrosion resistance performance (35°C, 100% humidity and 5.0 wt% NaCl solution). In addition, the obtained sample plates were also placed in acetic acid-salt spray testing chamber to evaluate the acetic acid-salt fog corrosion resistance performance (35°C, 100% humidity and 5.0 wt% NaCl solution, pH = 2.8 ~ 3.0).

## Results and discussion

### Preparation of CoAl_2_O_4_/Kaol hybrid pigments

**Figure 1:**
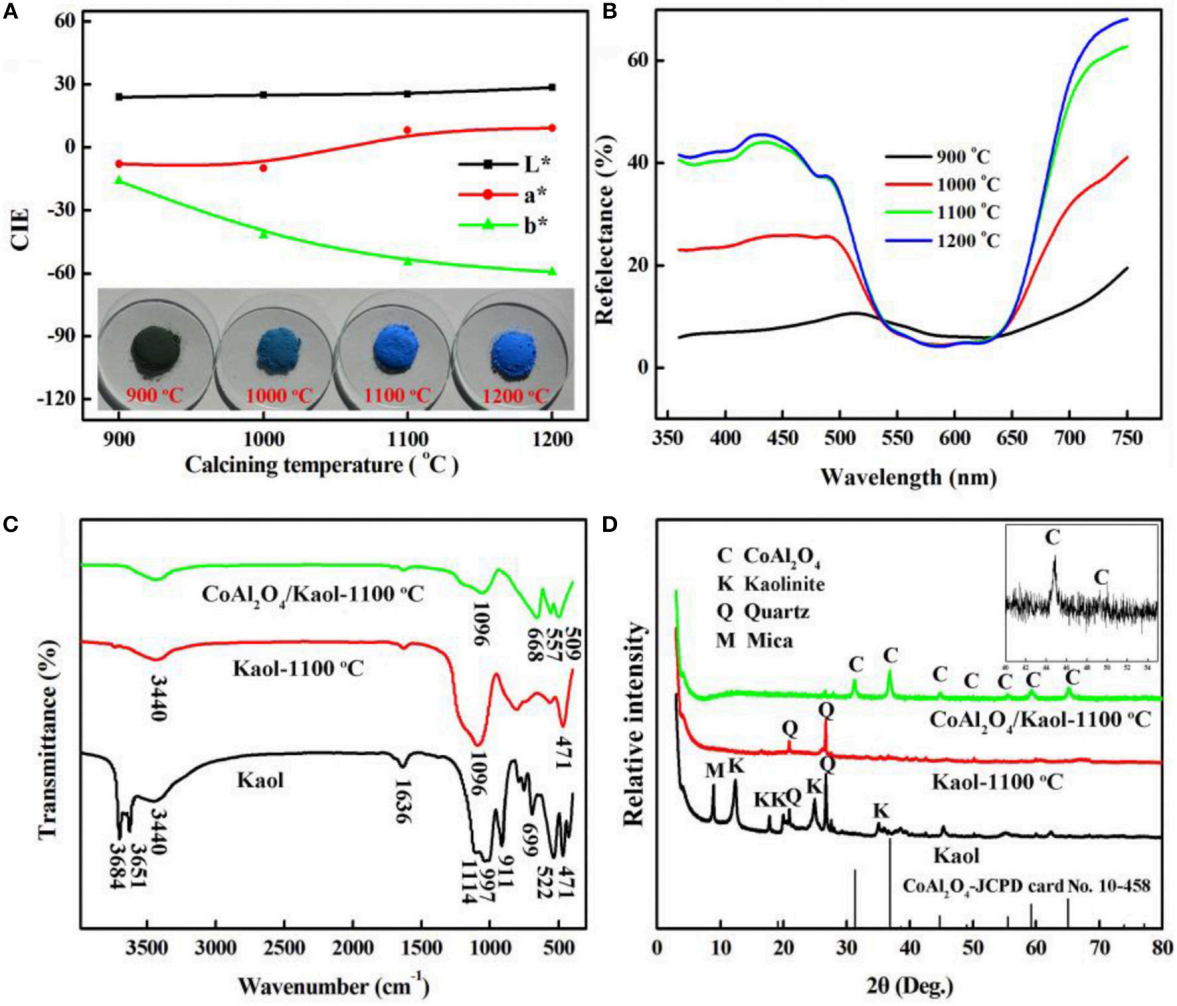
**(A)** CIE parameters and **(B)** reflectance spectra of CoAl_2_O_4_/Kaol hybrid pigments calcined at different temperatures, **(C)** FTIR, and **(D)** XRD patterns of Kaol, Kaol-1100°C, and CoAl_2_O_4_/Kaol-1100°C (the insert is the partial enlarged view of XRD patterns).

The calcining temperature is an important factor to form spinel CoAl_2_O_4_ with prefect blue color (Yang et al., 2013), and thus the effect of calcining temperatures on the color parameters of hybrid pigment was studied. Figure 1a gives the color parameters of CoAl_2_O_4_/Kaol hybrid pigments after being calcined at 900~1,200°C. The b^*^ value of hybrid pigments firstly decreases with the increase in the temperatures, and then it almost has no obvious change as the temperature is above 1,100°C. The values of L^*^ and a^*^ continuously increase with the increase in the calcining temperatures. With the increase in the calcining temperature from 900 to 1,100°C, the color of the hybrid pigments transforms from green to blue to bright blue, which is also consistent with the change of their color parameters. The blue color is derived from Co^2+^ in tetrahedral sites, while the green color is attributed to Co^3+^ in octahedral coordination (Álvarez-Docio et al., 2017). By contrast, the b^*^ value of hybrid pigments reaches the minimum value after being calcined at 1,100°C, suggesting that the optimum calcining temperature is 1,100°C. As shown in Figure 1b, the reflection bands and absorption bands are observed at 440~500 nm and at 550~650 nm, respectively, except for hybrid pigment prepared at 900°C. These bands are associated with the blue color of CoAl_2_O_4_ due to ^4^*A*_2_ (*F*) → ^4^*T*_1_ (*P*) of the Co^2+^ *d*-*d* transition in tetrahedral coordination (Tielens et al., 2006; Kurajica et al., 2012; Gomesan et al., 2015; Tahereh et al., 2016). And for the hybrid pigment prepared at 900°C, the bands at 380 and 670 nm are indicative of the occurrence of octahedrally coordinated Co^3+^, and are ascribed to the ^1^*A*_1*g*_→^1^*T*_2*g*_ and ^1^*A*_1*g*_→^1^*T*_1*g*_ transitions of low spin Co^3+^ in octahedral symmetry (Brik et al., 2001; Herrero et al., 2007), the sample exhibits greenish hue annealed at low temperature, which is also in accordance with the digital photos of the hybrid pigment calcined at 900°C (Kurajica et al., 2012). In addition, the intensity of the reflection peaks also increases with the increase in the preparation temperatures until 1,100°C, and then it decreases as the calcining temperature reaches 1,200°C, which may be due to crystal phase and structural transformation of Kaol (Juneja et al., 2010; Yeo, 2011).

Figure S1a depicts the effect of grinding time on color parameters of hybrid pigment, the b^*^ value of CoAl_2_O_4_/Kaol hybrid pigments firstly decreases with the increase in the grinding time from 0.5 to 1 h, and no obvious change is observed with the continuous increasing grinding time. In order to ensure the reaction uniformity, the grinding time is selected to 2 h. Figure S1b exhibits the effect of the added amount of Kaol on color parameters. At the same calcining temperature (1,100°C), the L^*^ value of hybrid pigments gradually increases with increase in the added amount of Kaol, while the a^*^ and b^*^ values of the sample containing Kaol of 35.5 wt% (1 g Kaol) reach maximum. Interestingly, CoAl_2_O_4_/Kaol hybrid pigments still exhibits good color properties (L^*^ = 29.9, a^*^ = 2.01, b^*^ = −54.93) as the added amount of Kaol is up to 81.5 wt% (8 g Kaol). However, the sample is atrovirens prepared without Kaol at 1,100°C, suggesting that the introduction the low-cost Kaol not only reduces the production cost of CoAl_2_O_4_, but also decreases the high temperature crystallization temperature for formation of spinel structure. During calcining process, Kaol (2SiO_2_·Al_2_O_3_·2H_2_O) transforms into metakaolinite (2SiO_2_·Al_2_O_3_) at 400~600°C, and then it turns into SiAl_2_O_5_ and amorphous SiO_2_ (>900°C), even amorphous SiO_2_ and low-order crystalline α-Al_2_O_3_ at above 1,100°C (Ribeiro et al., 2005; Gilkes and Prakongkep, 2016). Because the mass transfer process is the rate controlling step during CoAl_2_O_4_ preparation (He and Becker, 1997), the uniform distribution Co_3_O_4_ and Al_2_O_3_ on the surface of Kaol after being grinded is in favor of reducing the mass transfer resistance, thus the incorporation of Kaol decreases the calcining temperature for formation of spinel CoAl_2_O_4_ (Wang et al., 2006; Gabrovska et al., 2014). Based on above results, the optimum conditions for preparing hybrid pigment with the prefect color are 1,100°C and 81.5 wt% of Kaol.

### Characterization of CoAl_2_O_4_/Kaol hybrid pigment

Figure 1c presents FTIR spectra of Kaol, the calcined Kaol at 1,100°C and CoAl_2_O_4_/Kaol hybrid pigment, respectively. FTIR spectrum of Kaol exhibits the characteristic bands of Si-O-Si (at about 471, 522, 699, 1,032, and 1,114 cm^−1^), Si-O-Al (at around 525, 750, and 795 cm^−1^), Al-Al-OH (at around 522 and 911 cm^−1^), -OH (at about 3,684, 3,684, 3,651, and 3,440 cm^−1^), while the bending vibration of the inter-layer water is observed at 1,636 cm^−1^ (Rekik et al., 2017; Sreelekshmi et al., 2017). After being calcined, these typical absorption bands disappear compared with that of the raw Kaol, which is ascribed to the dehydroxylation and the crystal phase transition of Kaol during calcining process. In the case of FTIR spectrum of hybrid pigment, several new absorption bands appear at 668, 557, and 509 cm^−1^, which can be attributed to the stretching vibration of Al-O of AlO_6_ and Co-O of CoO_4_, respectively (Zayat and Levy, 2000; Zhang A. J. et al., 2017), indicating the formation of CoAl_2_O_4_.

Figure 1d shows the XRD patterns of Kaol, the calcined Kaol at 1,100°C and CoAl_2_O_4_/Kaol hybrid pigment, respectively. It is clear that the associated minerals are quartz (*2*θ = 20.8° and 26.7°) and mica (*2*θ = 8.9°) (Chhikara et al., 2015; Mymrin et al., 2017), and the typical characteristic peaks of kaolinite presents the well-defined reflections at 2θ = 12° and 25°. When Kaol was calcined at 1,100°C, only diffraction peaks of quartz are remained. For hybrid pigment, new diffraction peaks are observed at *2*θ = 31.1°, 36.8°, 44.8°, 49.0°, 55.5°, 59.2° and 65.2°, which correspond to (220), (311), (400), (331), (422), (511), and (440) planes of CoAl_2_O_4_ according to JCPD card No. 10-458, respectively (Xi et al., 2012; Zhu et al., 2015). In fact, CoO also reacts with silica derived from Kaol to form cobaltous silicate, but this reaction is considered to be a minor reaction since most of CoO react with Al_2_O_3_ because CoO has high affinity to Al_2_O_3_ (Ahmed et al., 2012).

Figure S2 shows Raman spectra of CoAl_2_O_4_ pigment without Kaol and CoAl_2_O_4_/Kaol hybrid pigment. CoAl_2_O_4_ pigment with a spinel structure usually exhibits five Raman active modes: *A*_1*g*_ (764 cm^−1^), *F*_2*g*_ (644, 511, and 203 cm^−1^) and *E*_*g*_ (413 cm^−1^). Hybrid pigment also clearly presents the five expected Raman active modes at 760, 641, 510, 410, and 202 cm^−1^, indicating that hybrid pigments is assigned to the spinel structure (Zha et al., 2016; Liu et al., 2017). Compared with the Raman spectrum of CoAl_2_O_4_, all modes of hybrid pigment shift to lower frequencies, and this red shift suggests that primary coordinative environments of tetrahedron and octahedron positions may change after incorporation of Kaol.

**Figure 2:**
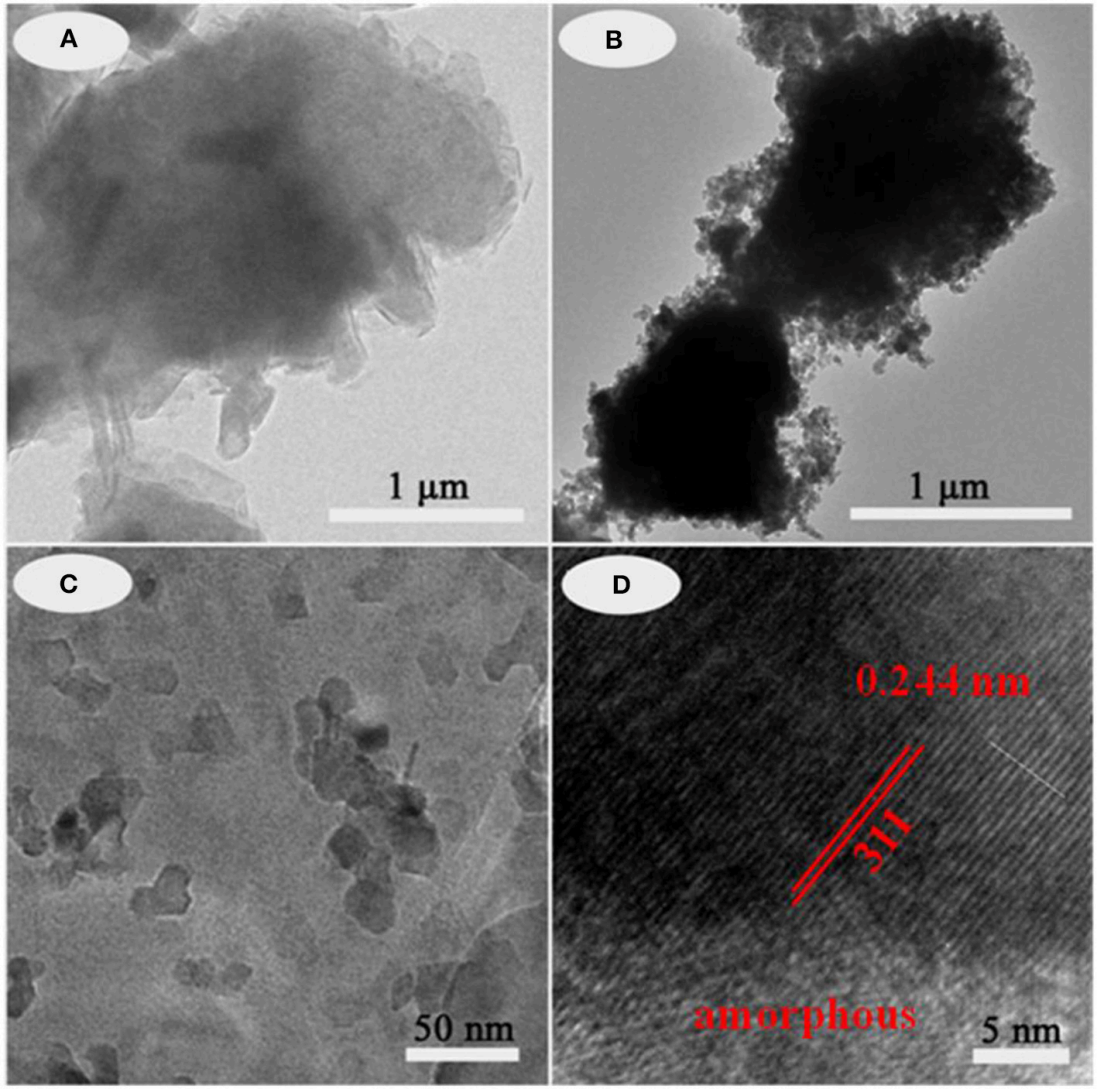
TEM images of **(A)** Kaol and **(B–D)** CoAl_2_O_4_/Kaol-1100°C.

**Figure 3:**
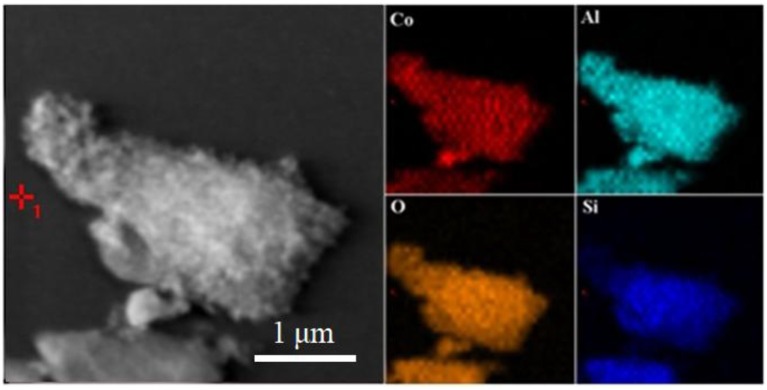
TEM image and the corresponding element mapping of CoAl_2_O_4_/Kaol-1100°C.

The typical structures and the morphology evolution of the samples can be observed by TEM. As illustrated in Figure 2a, Kaol presents a typical lamellar structure with a smooth surface. After being calcined at 1,100°C (Figure S3a), the lamellar morphology remains well, which reveals that Kaol possesses the good thermal stability. With the introduction of CoAl_2_O_4_ nanoparticles, the surface of lamellar morphology becomes coarse (Figures 2b,c), and CoAl_2_O_4_ nanoparticles are uniformly distributed on the substrate surface with a diameter of 10~20 nm. Furthermore, no obvious aggregation is observed, thus it indicates that incorporation of Kaol effectively prevents from the aggregation and controls the size of CoAl_2_O_4_ nanoparticles. The selected area electron diffraction pattern of hybrid pigment also indicates the successful formation of spinel CoAl_2_O_4_ (Figure S3b) (Naderi et al., 2014; Zou and Zheng, 2016). Figure 2d gives an enlarged electron micrograph, and it provides a well-resolved lattice plane with an interplanar spacing of 0.244 nm, corresponding to (311) plane of the cubic *Fd3m* space group, which is identified on the basis of data of the standard CoAl_2_O_4_ database JCPD card No. 10-458 (Kim et al., 2012). Meanwhile, the micrograph also displays the coexistence of amorphous and crystalline phases, which is attributed to the amorphous SiO_2_ derived from Kaol and spinel CoAl_2_O_4_, respectively (Cho and Kakihana, 1999). In addition, Figure 3 illustrates the elemental mapping of hybrid pigment, it is clear that hybrid pigment is mainly composed of Co, Al, O, and Si elements. Co element is uniformly distributed on the surface of lamellar substrates, suggesting that the generated CoAl_2_O_4_ is uniformly anchored on the surface of substrate.

**Figure 4:**
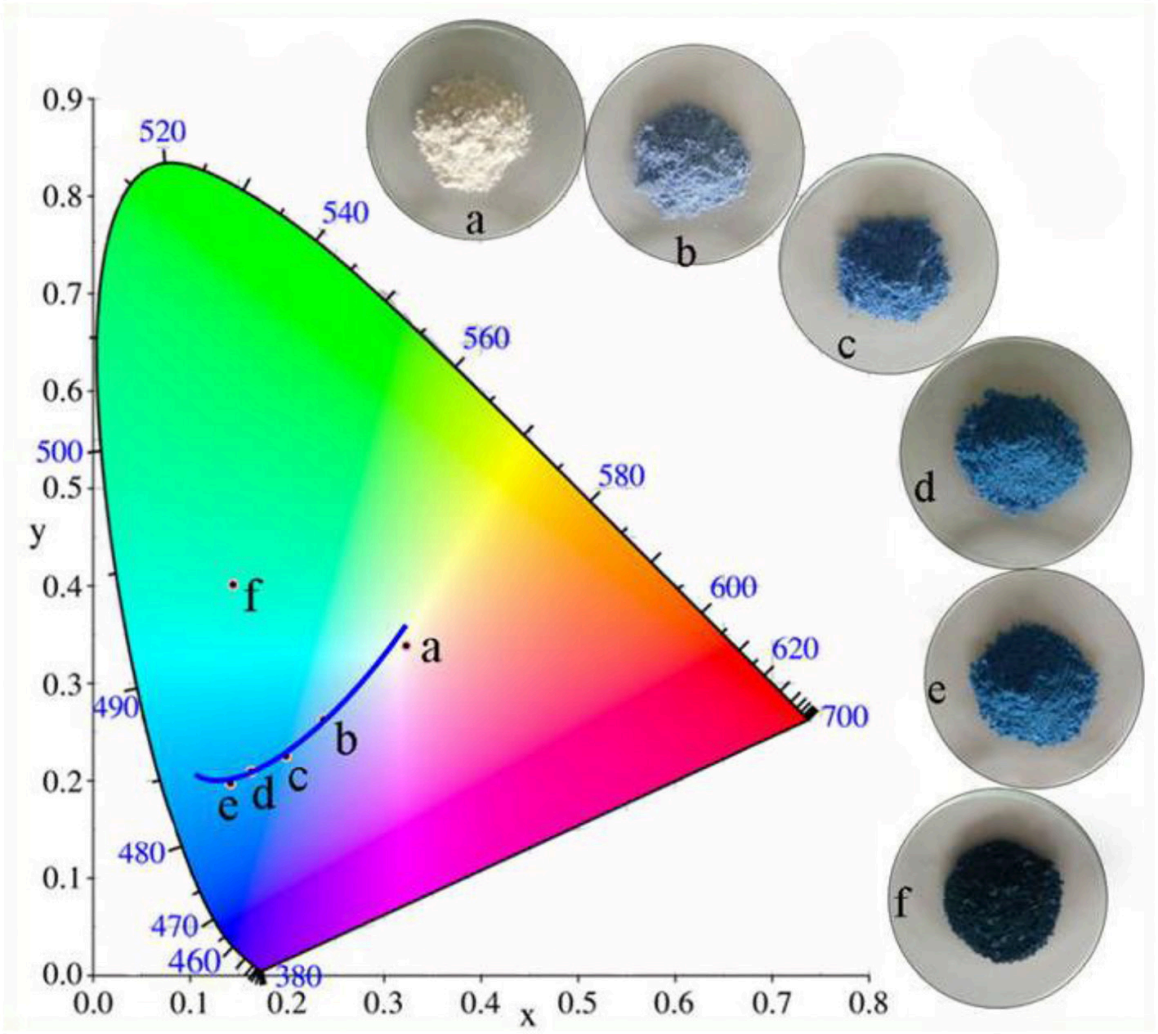
Color coordinates of pigment prepared using the different addition of Co_3_O_4_ in the presence of 2 g Kaol without Al_2_O_3_ (a-0%, b-2.4%, c-4.8%, d-9.1%, e-13%, f-16.7%).

Based on our previous studies, Kaol is not merely a carrier for loading of CoAl_2_O_4_ nanoparticles, Al_2_O_3_ as one of the main compositions of Kaol might participate in the high-temperature crystallization to form clay mineral doped spinel CoAl_2_O_4_. In order to confirm above inference, the sample was prepared using Co_3_O_4_ and Kaol without Al_2_O_3_ by the same procedure with CoAl_2_O_4_/Kaol hybrid pigments. As shown in Figure 4 and Table S2, the color of the as-prepared pigment is blue, and the values of L^*^ and a^*^ decrease with the increase of the addition amount of Co_3_O_4_, but b^*^ firstly decrease with the increase of the added amount of Co_3_O_4_ to 13%, and then it begins to increase as Co_3_O_4_ reaches 16%. At this moment, the obtained pigment is dark-green color, which is mainly ascribed to the fact that the excess Co_3_O_4_ is not fully involved in the reaction and anchors on the pigment surface (Aguilar-Elguézabal et al., 2017). At the low addition amount of Co_3_O_4_ (4.8%), CoO derived from the thermal reduction of Co_3_O_4_ can react with Al_2_O_3_ derived from Kaol to form spinel CoAl_2_O_4_ at 1,100°C, which can be confirmed by their XRD patterns and FTIR spectra (Figure S4). Therefore, it can be safely concluded that Kaol may be served as an aluminum source to take part in the crystallization process of CoAl_2_O_4_.

### Possible formation mechanism of CoAl_2_O_4_/Kaol hybrid pigment

**Scheme 1:**
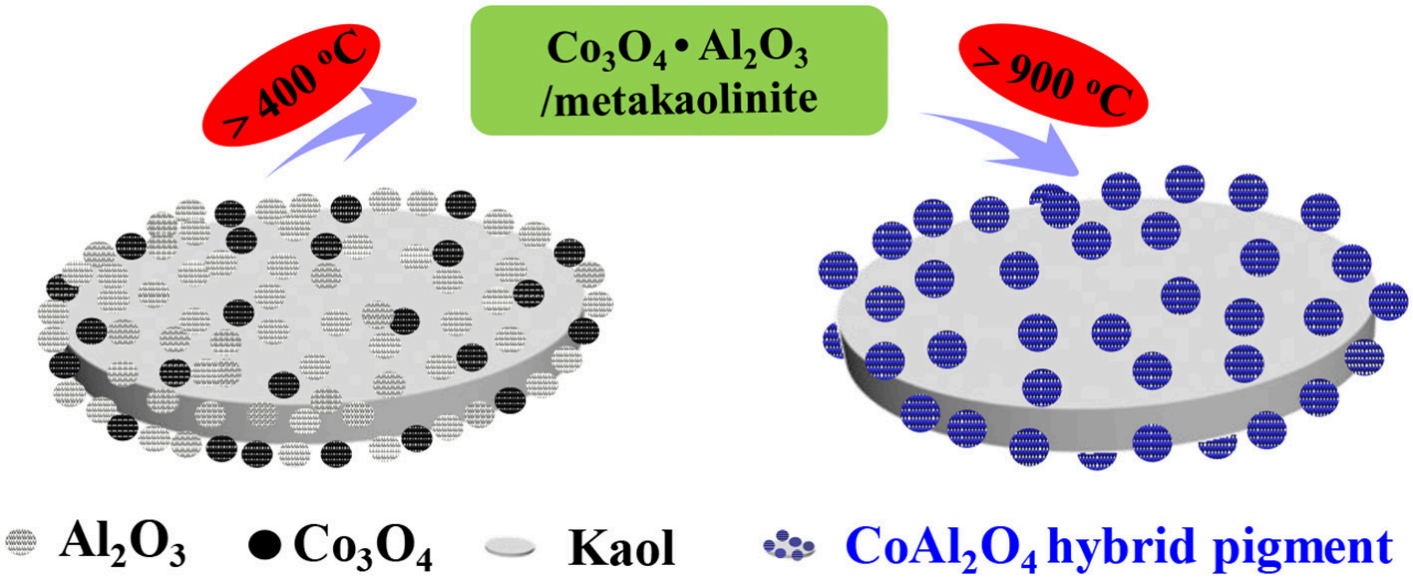
Possible formation mechanism of CoAl_2_O_4_/Kaol hybrid pigment.

According to the above results, the as-prepared hybrid pigment exhibit good color performances at a low Co content compared with the commercial CoAl_2_O_4_ pigments even 7 wt%. The possible formation mechanism of CoAl_2_O_4_/Kaol hybrid pigments can be described as follows: Co_3_O_4_ and Al_2_O_3_ can be uniformly distributed on the surface of Kaol during grinding. With the increase of the calcining temperature (400~600°C), Kaol (2SiO_2_·Al_2_O_3_·2H_2_O) is turned into metakaolinite (2SiO_2_·Al_2_O_3_). As the calcining temperature increases to 900°C, Co_3_O_4_ begins to thermally reduce into CoO, subsequently the increase in the calcining temperature results in the formation of spinel CoAl_2_O_4_ based on the reaction between CoO and Al_2_O_3_ on Kaol substrate accompanied with the crystal phase transition of Kaol to amorphous SiO_2_ and low-order crystalline α-Al_2_O_3_. In this process, the uniform distribution of Co_3_O_4_ and Al_2_O_3_ may be expected to decrease the mass transfer resistance for formation of spinel CoAl_2_O_4_, which reduces the processing time and calcining temperature, while the traditional solid-phase method for preparation of CoAl_2_O_4_ spends the long time ranging from several hours to days at high temperature (>1,200°C) (Wang et al., 2006). Furthermore, α-Al_2_O_3_ from Kaol also simultaneously reacts with CoO during high-temperature crystallization, thus the generated CoAl_2_O_4_ and the calcined production of Kaol finally form a solid solution. The possible formation mechanism of CoAl_2_O_4_/Kaol hybrid pigment can be illustrated in Scheme 1.

### Environmental stability of CoAl_2_O_4_/Kaol hybrid pigments

As shown in Figure S5a, the reflectance spectra of hybrid pigments are nearly same before and after being exposed under UV light for 15 days, indicating the excellent stability of hybrid pigment to UV light. In addition, the color of the sample plates has no obvious difference before and after being immersed into HCl, NaOH and ethanol, suggesting the excellent resistance of hybrid pigments to the corresponding reagents, which also can be confirmed from their color parameters (Table 2). Meanwhile, CoAl_2_O_4_/Kaol hybrid pigment presents the excellent thermal stability (Figures S5b,c).

**Table 2:** Color parameters of the sample plates before and after being immersed into 3M HCl, 3M NaOH, and ethanol for 72 h, respectively.

**Different medium**	**Color parameters**		
	L^*^	a^*^	b^*^
Before immersion	43.68	−7.88	−59.33
3M HCl	42.86	−7.56	−59.38
3M NaOH	43.55	−7.63	−59.12
Ethanol	43.83	−7.92	−59.34

The Commission Internationale de l'Eclairage (CIE) 1976 L^*^,a^*^,b^*^ colorimetric method was revised based on the Hunter L^*^a^*^b (1958). And the parameters of L^*^ is the lightness axis (0 for black and 100 for white). The parameters of a^*^ (negative values for green and positive values for red) and b^*^(negative values for blue and positive values for yellow) denote the hue or color dimensions.

In this study, the potential applications of the as-prepared hybrid pigment were also explored in this study. The thermal resistance coating was prepared by following procedures: Firstly, 5 wt% CoAl_2_O_4_/Kaol hybrid pigment was dispersed into ethyl acetate and stirred under high shear for 30 min (2,000 rpm), and the above obtained mill base were added into commercial thermal resistance coating and stirred under low shear for 60 min (500 rpm). The viscosity of the coating was adjusted by ethyl acetate to 20~30 s. And then it was sprayed onto steel plate substrate of size 15 cm × 10 cm × 0.1 cm and dried under the standard conditions (temperature: 25°C and humidity: 40%) for 7 days (the coating thickness: D = 50 ± 2 μm). The obtained sample plates were shown in Figure S6c, it is clear that the surface of the sample plate is smooth after hybrid pigment (5 wt%) is added into the commercial thermal resistance coating, indicating hybrid pigment disperses well in the painting. Furthermore, the film containing hybrid pigments exhibits the similar color parameters (L^*^ = 24.88, a^*^ = 13.54, b^*^ = −57.80) (Figure S6d) after being calcined at 800°C for 2 h. However, the steel plate coated the commercial thermal resistance coating (Figure S6a) is damaged heavily (Figure S6b). It indicates that the film containing hybrid pigment has a good thermal stability, and it can effectively protect steel plate substrate to avoid excessive damage. Meanwhile, this film also exhibited excellent fireproof properties, which can be illustrated by its fire test using a flame gun (800~1,000°C) (Figure S6e). It is found that this film has no obvious change after being placed on the fire for 30 s (Movie S1 and Figure S6f), revealing that the hybrid pigment may be used as fireproof paint. In addition, the as-prepared hybrid pigment also can be used as a colored painting and artistic pigment (Figures S7a,b).

### Anticorrosion coating application of hybrid pigments

**Figure 5:**
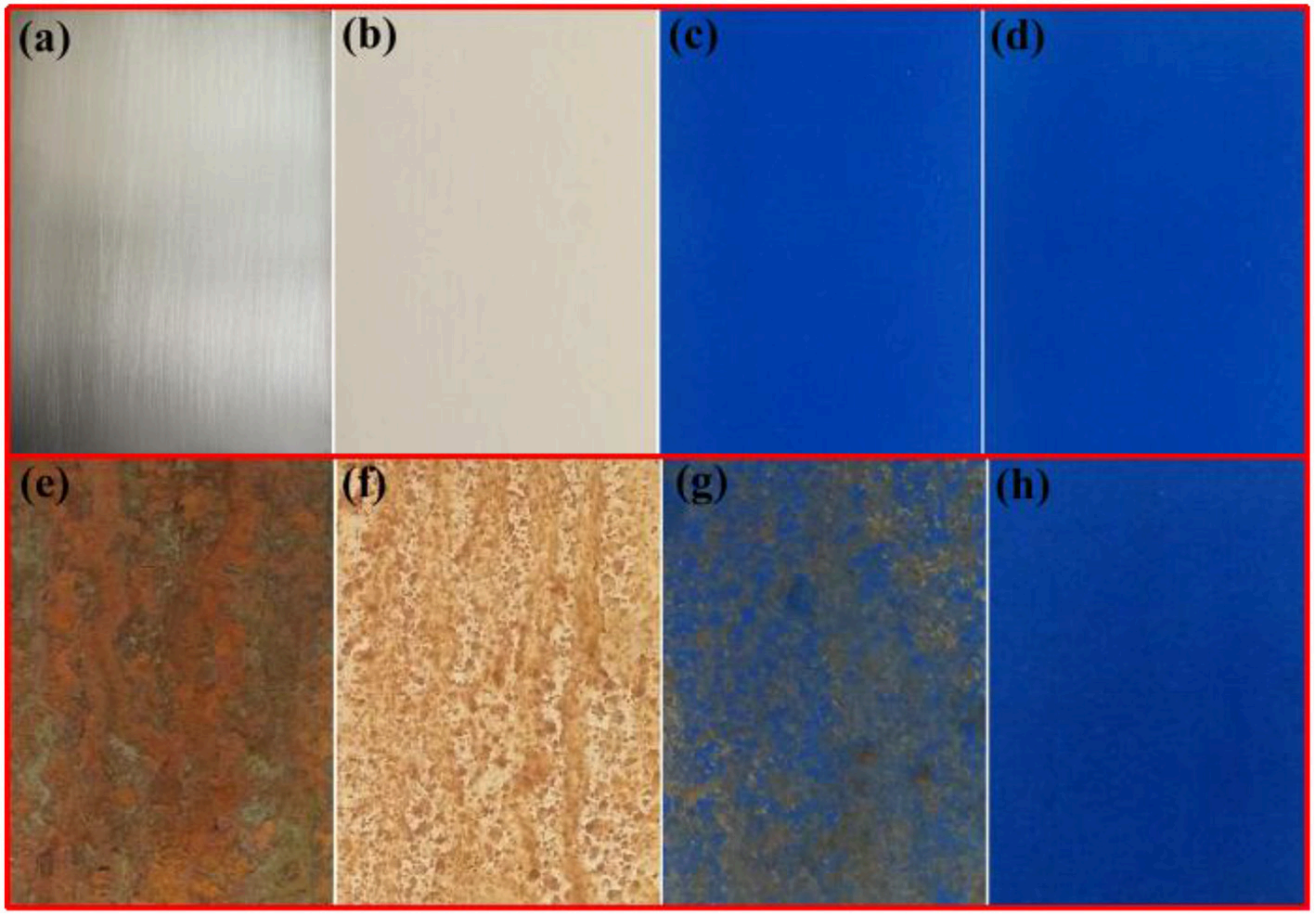
Photographs of **(a)** steel substrate, **(b)** steel coated the paint without pigment, **(c)** steel coated the paint containing commercial CoAl_2_O_4_, **(d)** steel coated the paint containing hybrid pigment. **(e–h)** Their corresponding images after being exposed to salt spray test for 60 days, respectively.

**Scheme 2:**
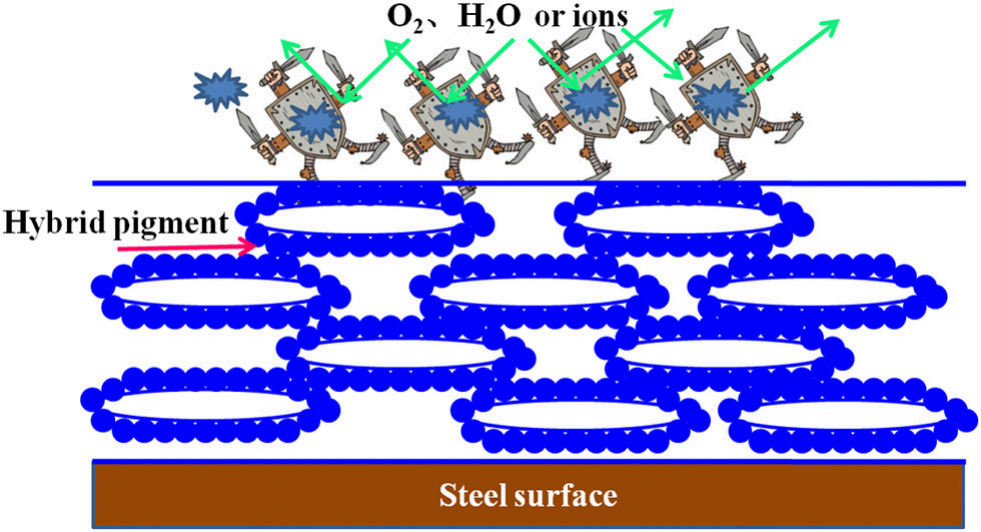
The corrosion protection mechanism of the anticorrosion coating.

Corrosion is a natural and gradual destruction process of metal materials resulting from chemical and/or electrochemical reaction with their environment, which leads to enormous losses to our lives, finances, etc. Therefore, corrosion engineering is the field to control and stop corrosion using various methods, especially surface coating protection technology, in which inorganic pigments (e.g., ZnO and TiO_2)_ are commonly incorporated to improve the anticorrosion properties of coating, and perhaps provide the color for coating (e.g., α-Fe_2_O_3_). Therefore, the as-prepared hybrid pigment is incorporated into the common epoxy anticorrosion coating to investigate its anticorrosion properties. As shown in Figure 5, the steel plate substrate suffers from an aggravating corrosion referred from the large-scale red rust without the epoxy anticorrosion coating (Figure 5e). After coating of epoxy anticorrosion coating, the corrosion of steel plate is slightly improved (Figure 5f). In particular, this anticorrosion effect is obvious after introducing the commercial CoAl_2_O_4_ pigments (Figure 5g). With the incorporation of the as-prepared hybrid pigment, no obvious rust was found on the surface of coating (Figure 5h). It suggests that the introduction of hybrid pigment obviously improves the corrosion resistance of coating. This is mainly ascribed to the synergistic effect between the lamellar layered structure of Kaol and the excellent weatherability of CoAl_2_O_4_, which can prevents small molecules (H_2_O and O_2_) or ions from penetrating into the coating film. Thus, the relevant mechanisms of anticorrosion can be illustrated in Scheme 2.

**Figure 6:**
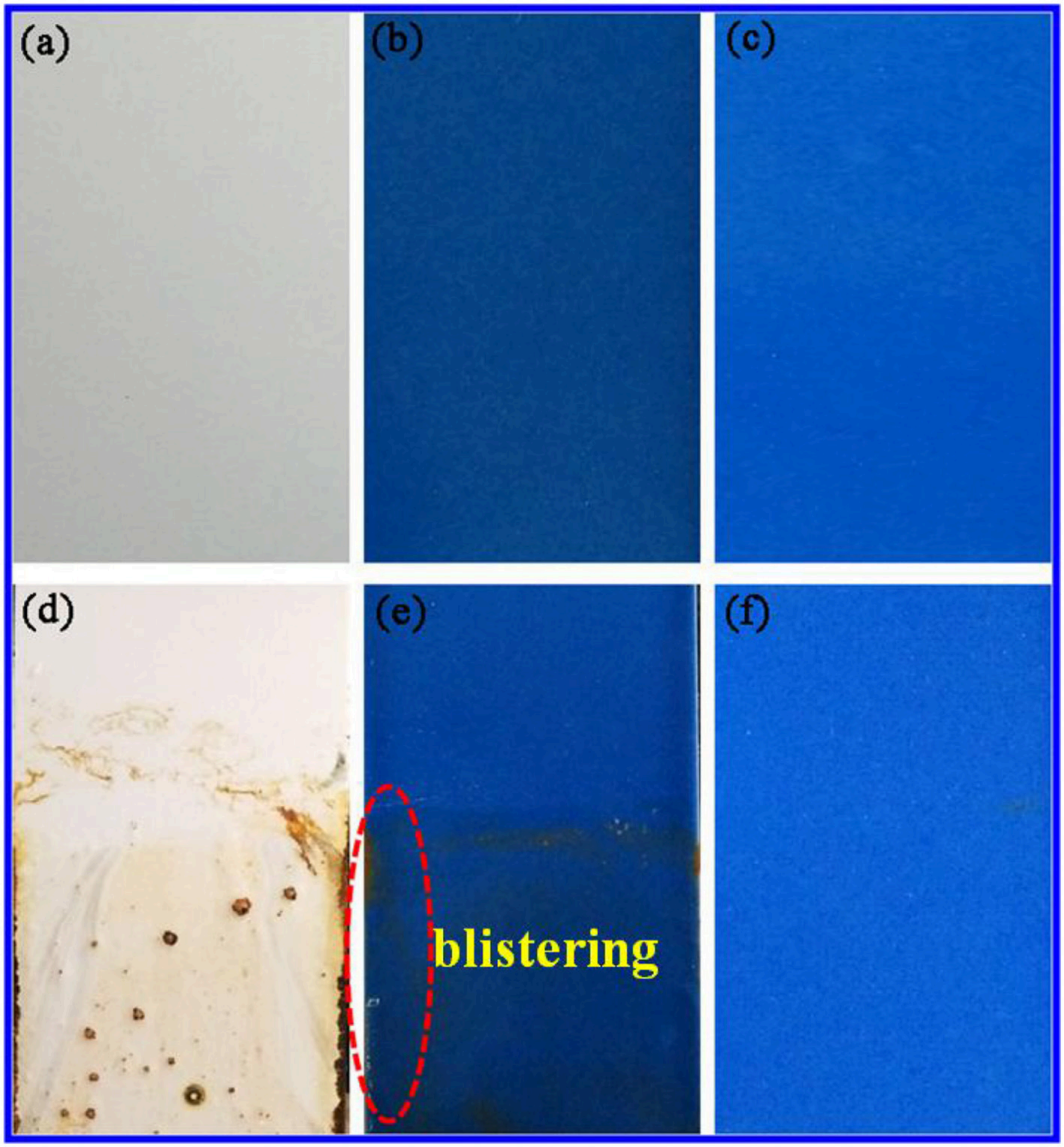
Photographs of the film **(a)** without pigment, **(b)** containing CoAl_2_O_4_, and **(c)** hybrid pigment. **(d–f)** Their corresponding images after being exposed to 3.5% NaCl for 60 days, respectively.

Furthermore, the chemical stability of the anticorrosion coating film was also evaluated by immersing into 3M HCl, 3M NaOH, and ethanol at room temperature for 72 h, respectively. The film has no obvious difference before and after being immersed, exhibiting the excellent resistance to HCl, NaOH, and ethanol (Figure S8). This is also confirmed by their color parameters before and after being immersed in the corresponding reagents (Table 3). More importantly, the obtained sample plates were also immersed into 3.5 wt% saline water to study the salted water resistance test for 60 days. As shown in Figure 6, the surface of coating film without pigment is smooth and dense before immersing (Figure 6a). After adding of CoAl_2_O_4_ without Kaol, the film color seems darker compared with that of containing CoAl_2_O_4_/Kaol hybrid pigment (Figures 6b,c). As expected, the film without pigment is swelling, blistering, and rusty after immersed into saline water for 60 days (Figure 6d). Furthermore, the same phenomenon also is observed from the one containing CoAl_2_O_4_ (Figure 6e), but the situation is better than the former due to the good weatherability of CoAl_2_O_4_ to acid, salt, etc. Surprisingly, the film incorporating of CoAl_2_O_4_/Kaol hybrid pigment was perfect without any defects (Figure 6f). It suggests that the coating film exhibited the excellent salted water resistance, which may be ascribed to the synergistic effect of the lamellar layered structure of Kaol and the excellent weatherability of CoAl_2_O_4_.

**Table 3:** Color parameters of the film before and after being immersed into 3M HCl, 3M NaOH and ethanol for 72 h, respectively.

**Different medium**	**Color parameters**		
	L^*^	a^*^	b^*^
Before immersion	25.11	13.97	−58.10
3M HCl	25.12	13.86	−58.05
3M NaOH	24.88	13.75	−57.88
Ethanol	25.13	13.88	−58.31

The Commission Internationale de l'Eclairage (CIE) 1976 L^*^,a^*^,b^*^ colorimetric method was revised based on the Hunter L^*^a^*^b (1958). And the parameters of L^*^ is the lightness axis (0 for black and 100 for white). The parameters of a^*^ (negative values for green and positive values for red) and b^*^(negative values for blue and positive values for yellow) denote the hue or color dimensions.

**Figure 7:**
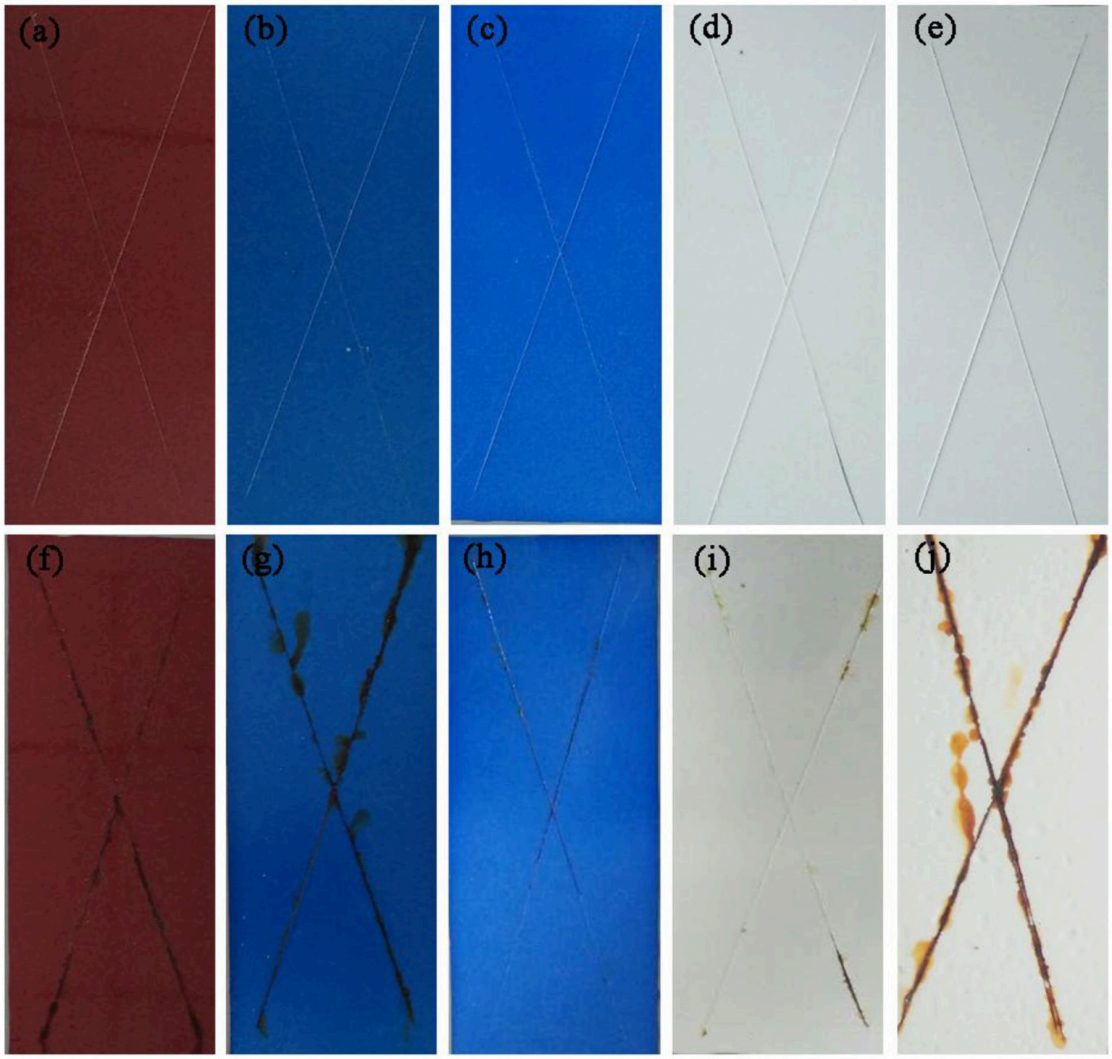
Photographs of the film containing **(a)** Fe_2_O_3_, **(b)** CoAl_2_O_4_, **(c)** CoAl_2_O_4_/Kaol hybrid pigment, **(d)** Kaol, and **(e)** ZnO. **(f–j)** The corresponding images of the film after being exposed to acetic acid-salt spray for 60 days, respectively.

**Figure 8:**
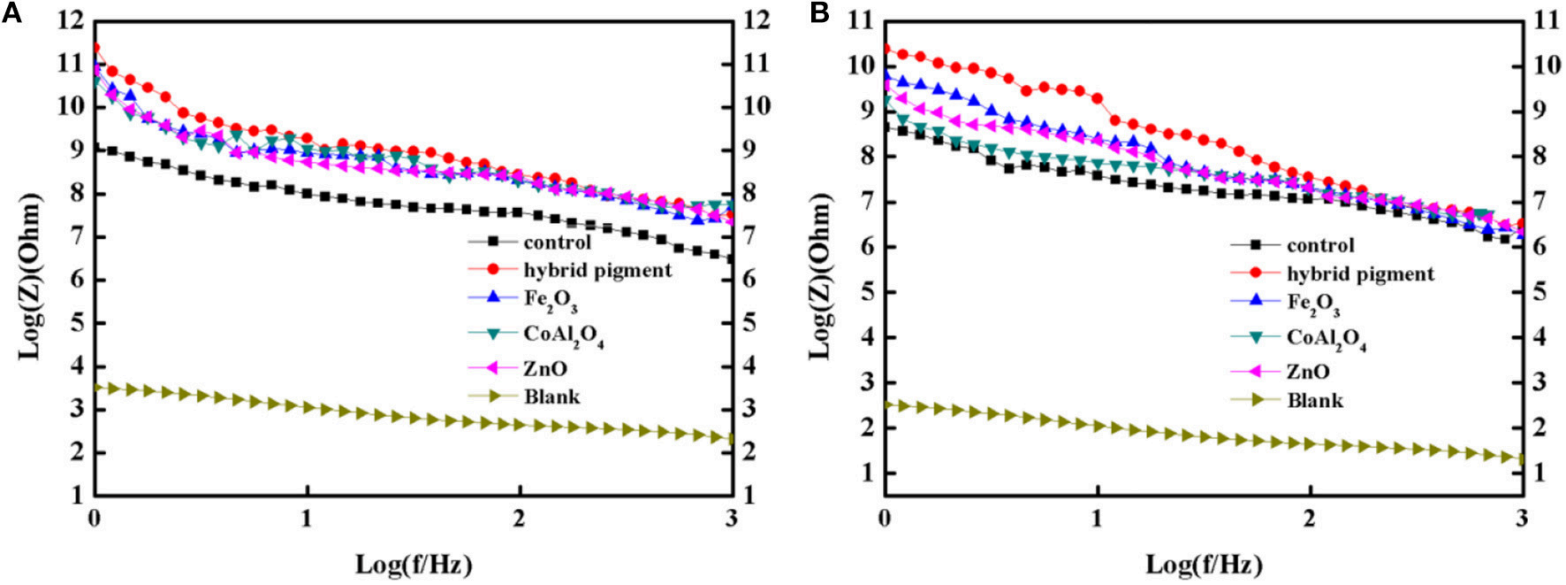
Bode plots of the coatings containing CoAl_2_O_4_/Kaol hybrid pigment, ZnO, Fe_2_O_3_, CoAl_2_O_4_, the control without pigment and steel before **(A)** and after **(B)** being immersed in the 3.5 wt% NaCl solution for 60 days.

Furthermore, the obtained sample plate containing hybrid pigment presents the better acid resistance than that of incorporating of ultramarine after contacting with 36.5 wt% HCl, which changes from blue to white due to its instability to acid (Figure S9). Therefore, the obtained sample plate was also placed in acetic acid-salt spray testing chamber to evaluate the acetic acid-salt fog corrosion resistance of the anticorrosion coating. As a control, the coatings containing Kaol, ZnO, Fe_2_O_3_ and CoAl_2_O_4_ calcined at 1,100°C are also investigated, and the components of paints were provided in Table S3. Firstly, the different amounts of the CoAl_2_O_4_/Kaol hybrid pigment were added into epoxy resin to determine the amounts of the hybrid pigment, and the results are shown in Figure S10. It is clear that the sample that containing 5 wt% CoAl_2_O_4_/Kaol hybrid pigment is bright blue, so the added amounts of the hybrid pigment is 5 wt%. And then the sample containing Kaol, ZnO, Fe_2_O_3_, and CoAl_2_O_4_ calcined at 1,100°C are shown in Figure 7, the corrosion of the films containing Fe_2_O_3_ (Figure 7f), CoAl_2_O_4_ calcined at 1,100°C (Figure 7g), and ZnO (Figure 7j) are very serious after be exposed in salt spray testing chamber 60 days. Interestingly, the corrosion of the sample containing CoAl_2_O_4_/Kaol hybrid pigment (Figure 7h) and Kaol (Figure 7j) were negligible, especially the sample of hybrid pigment. It may be ascribed to the excellent acid resistance and lamellar layered structure of hybrid pigment and Kaol, respectively. In addition, Figure 8 gives the anticorrosion ability of the coatings containing CoAl_2_O_4_/Kaol hybrid pigments, ZnO, Fe_2_O_3_, CoAl_2_O_4_ calcined at 1,100°C and the one without pigment. Bode plots indicates that the addition of CoAl_2_O_4_/Kaol hybrid pigments in epoxy paint shows a significant improvement in the anticorrosion properties compared with the pure epoxy paint and others pigment. It also suggests the synergistic effect between the lamellar layered structure of Kaol and the excellent weatherability of CoAl_2_O_4_ is in favor of improving the resistance to salts, which also is superior to the reported works (Table 4).

**Table 4:** Comparison with the anticorrosion performance of the reported additives and hybrid pigment in epoxy anticorrosive coating.

**Condition**	**Materials (added amount)**	**Time/h**	**References**
Neutral salt spray test	H-BN decorated with Fe_3_O_4_ (2%)	720	Zhang et al., 2016
Neutral salt spray test	Graphite nanoplatelets (0.5%)	750	Mohammadi et al., 2014
Neutral salt spray test	Zn (6%)	500	Bagherzadeh and Mousavinejad, 2012
Neutral salt spray test	ZnO/nano-Co:ZnO (6%)	168	Rostami et al., 2014
Neutral salt spray test	ZnO (6.5%)	720	Naderi et al., 2014
Neutral salt spray test	Ceramic nanocontainers (4%)	750	Kartsonakis et al., 2012
Neutral salt spray test	Hybrid pigments (5%)	1,440	This work
Acetic acid-salt spray test	Hybrid pigments (5%)	1,440	This work

**Figure 9:**
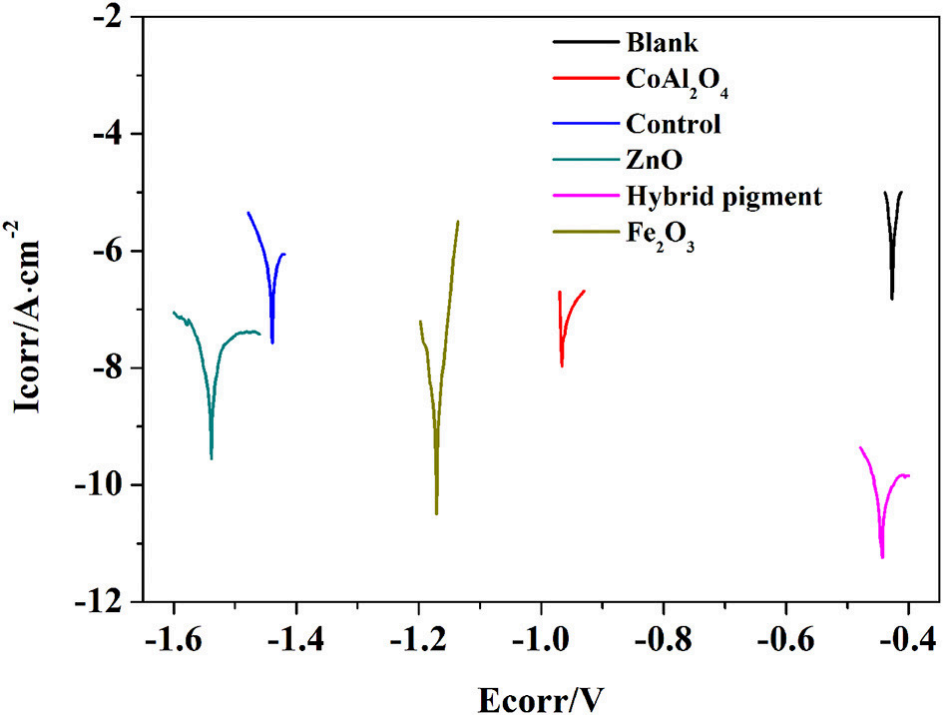
Tafel plots of blank and anticorrosion coatings containing CoAl_2_O_4_ calcined at 1,100°C, control without pigment, ZnO, CoAl_2_O_4_/hybrid pigment, and Fe_2_O_3_, respectively.

Anti-corrosion of the anticorrosive film is commonly determined from electrochemical measurements such as corrosion potential (Ecorr) and corrosion current (Icorr), and Electrochemical Impedance Spectroscopy (EIS) measurements were carried out with the use of an electrochemical working station (CHI660E) at open circuit potential. A three-electrode cell including the Ag/AgCl (3M KCl) reference electrode, the studied sample as working electrode and the platinum counter electrode was used to run EIS tests. EIS measurements are studied in the frequency range from 100 kHz to 0.005 Hz with a perturbation of 5 mV, and the working electrode area was 1 cm^2^. Figure 9 shows the Tafel plots generated for the anticorrosion coating containing CoAl_2_O_4_/hybrid pigment, ZnO, Fe_2_O_3_, CoAl_2_O_4_ calcined at 1,100°C, control (without pigment) and blank immersed in 3.5 wt% NaCl solution about 30 min. And the Tafel curve parameters are given in Table 5. As shown in Figure 9 and Table 5, the corrosion current of the control (without pigment) decreases from −1.52 × 10^−7^ A to −3.39 × 10^−8^ A compared with the blank, and the corrosion current obviously declines when the pigments were added into the anticorrosion coating. After incorporation of CoAl_2_O_4_/hybrid pigment, the corrosion current decreases from −3.39 × 10^−8^ A to −5.75 × 10^−12^ A, while the corrosion potential increases from −1.439 V to −0.443 V. In fact, the anticorrosion ability of the coating is related to the chemical composition and microstructure. The lower of the corrosion current or the higher corrosion potential suggests the better of the anticorrosion ability (Liang et al., 2007; Chang et al., 2012). Therefore, the anticorrosion coatings containing CoAl_2_O_4_/hybrid pigment, ZnO, Fe_2_O_3_ present good anticorrosion ability, and CoAl_2_O_4_/hybrid pigment exhibits superior anticorrosion ability compared with than that of ZnO and Fe_2_O_3_, which is consistent with that of the Bode plots (Figure 8), implying that the addition of CoAl_2_O_4_/Kaol hybrid pigments in epoxy paint shows a significant improvement in the electrochemical corrosion properties compared with the pure epoxy paint and other pigments.

**Table 5:** Tafel curve parameters of the anticorrosion coatings containing different pigments and blank sample.

**Sample**	**Corrosion potential (Ecorr)/V**	**Corrosion current density (Icorr)/A·cm^−2^**	**Corrosion current (I)/A**
Blank	−0.427	−6.82	−1.52 × 10^−7^
CoAl_2_O_4_	−0.966	−7.96	−1.10 × 10^−8^
Fe_2_O_3_	−1.171	−10.50	−3.16 × 10^−11^
ZnO	−1.538	−9.55	−2.82 × 10^−10^
Control	−1.439	−7.57	−3.39 × 10^−8^
Hybrid pigment	−0.443	−11.24	−5.75 × 10^−12^

## Conclusion

In conclusion, the low-cost bright blue CoAl_2_O_4_/Kaol hybrid pigments were successfully prepared by the traditional solid-state reaction. The introduction of Kaol not only reduces the Co consumption and the temperature for formation of spinel CoAl_2_O_4_, but also enhances the color properties of cobalt blue pigments with bright blue and high NIR reflectance, especially prepared using 8.1% Co_3_O_4_ and 81.5% Kaol. During preparation, Co_3_O_4_ and Al_2_O_3_ can be uniformly distributed on the surface of Kaol, which reduces the mass transfer resistance and prevents from the aggregation CoAl_2_O_4_ nanoparticles during high-temperature crystallization process. In addition, the Al_2_O_3_ originated from Kaol also may participate in the formation of spinel CoAl_2_O_4_ pigments. Due to the synergistic effect between the lamellar layered structure of Kaol and the excellent weatherability of CoAl_2_O_4_, the anticorrosion coating containing hybrid pigments exhibited the excellent corrosion resistance toward the salt and acetic acid-salt system. Furthermore, the CoAl_2_O_4_/Kaol hybrid pigments prepared by traditional solid-phase method can be applied in thermal resistance coating, wall painting and artistic pigment.

## Author contributions

AZ and XW contribute to the experiment process, samples characterization, data analysis, and paper preparation. BM and AW are mainly responsible for the design of experiment, data analysis, and paper revision.

### Conflict of interest statement

The authors declare that the research was conducted in the absence of any commercial or financial relationships that could be construed as a potential conflict of interest.
