# Supplementary material for: CoAl2O4/Kaoline Hybrid Pigment Prepared via Solid-Phase Method for Anticorrosion Application
Source: Front Chem. 2018 Nov 29;6:586. doi: 10.3389/fchem.2018.00586 (PMC6281987; doi:10.3389/fchem.2018.00586)
Supplement: Supplementary file 1 [file Data_Sheet_1.pdf]

$CoAl_2O_4$/ kaoline hybrid pigment prepared via solid-phase method for anticorrosion application

Anjie Zhang,[a,b] *Bin Mu,*[a*] *Xiaowen Wang,*[a,b] *and Aiqin Wang*[a*]

[a] Key Laboratory of Clay Mineral Applied Research of Gansu Province, Center of Eco-material and Green Chemistry, Lanzhou Institute of Chemical Physics, Chinese Academy of Sciences, Lanzhou 730000, PR China,

[b] University of Chinese Academy of Sciences, Beijing 100049, PR China.

*Corresponding authors. E-mail addresses: mubin@licp.cas.cn (B. Mu) and aqwang@licp.cas.cn (A. Wang); Fax: +86 931 8277088; Tel: +86 931 4868118.

Figure captions:

Figure S1. Relationship between CIE parameters of hybrid pigment prepared at different conditions: (a) different grinding time and (b) different addition of Kaol.

Figure S2. Raman spectra of $CoAl_2O_4$ pigment and $CoAl_2O_4$/Kaol hybrid pigment calcined at 1100 ℃.

Figure S3. (a) TEM image of Kaol calcined at 1100 ^{o}C and (b) the selected area electron diffraction pattern of hybrid pigment

Figure S4. (a) XRD pattern and (b) FTIR spectrum of the as-prepared sample prepared using 13% Co_3O_4 and Kaol in the absence of Al_2O_3.

Figure S5. (a) UV-vis reflectance spectra of $CoAl_2O_4$/kaoline hybrid pigment before and after being exposed in UV accelerated weathering tester for 15 days (the insert is the photographs of hybrid pigment before and after being exposed under UV light). (b) and (c) Digital photos of $CoAl_2O_4$/kaoline hybrid pigment before and after being calcined at 1000℃ .

Figure S6. Digital photos of steel plates coated the commercial thermal resistance paint (a and b), and the one containing hybrid pigment (c and d) before and after being calcined at 800°C for 2 h. The image of (e) flame gun and (f) the commercial thermal resistance paint containing hybrid pigment after being heated, respectively.

Figure S7. $CoAl_2O_4$/kaoline hybrid pigments prepared at different temperatures applied as a painting (a) and art pigment (b).

Figure S8. Digital photos of the sample plate immersed in 3 mol/L HCl, 3 mol/L H_2SO_4, 3 mol/L NaOH and ethanol after 72 h, respectively.

Figure S9. Digital photos of (a) ultramarine and (b) hybrid pigment after contacting with 36.5 wt% HCl.

Figure S10. Digital photos of the anticorrosion coatings containing (a) 1%, (b) 2%, (c) 3%, (d) 4%, (e) 5% hybrid pigment and (f) 5% $CoAl_2O_4$ pigment calcined at 1200°C, respectively.

Table S1 Chemical compositions of Kaol before and after being treated using HCl.

Table S2 CIE parameters of hybrid pigments prepared using 2 g Kaol and different contents of Co_3O_4 in the absence of Al_2O_3.

Table S3 Paint formulations

Movie S1 The film containing hybrid pigment being placed on the fire for fire test using a flame gun

Table S1 Chemical compositions of Kaol before and after being treated using HCl.

Kaol	Al_2O_3(%)	Na_2O(%)	MgO(%)	CaO(%)	SiO_2(%)	K_2O(%)	Fe_2O_3(%)
Before	27.45	0.17	0.52	0.07	43.12	1.3	0.18
After	27.43	0.23	0.38	0.01	48.71	3.55	0.37

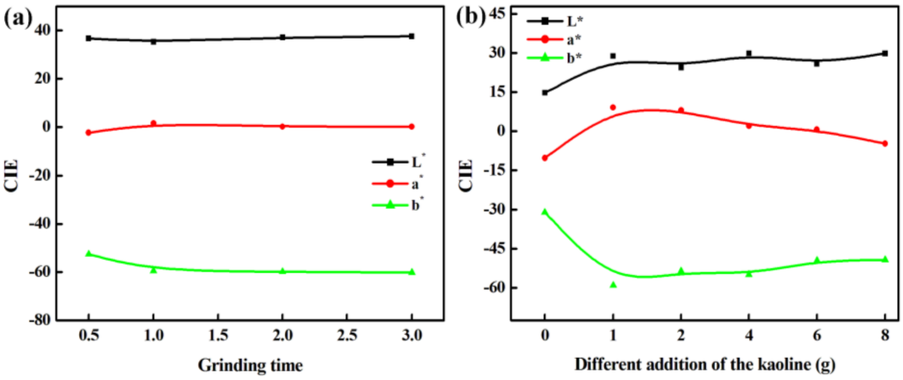

Figure S1. Relationship between CIE parameters of hybrid pigment prepared at different conditions: (a) different grinding time and (b) different addition of Kaol.

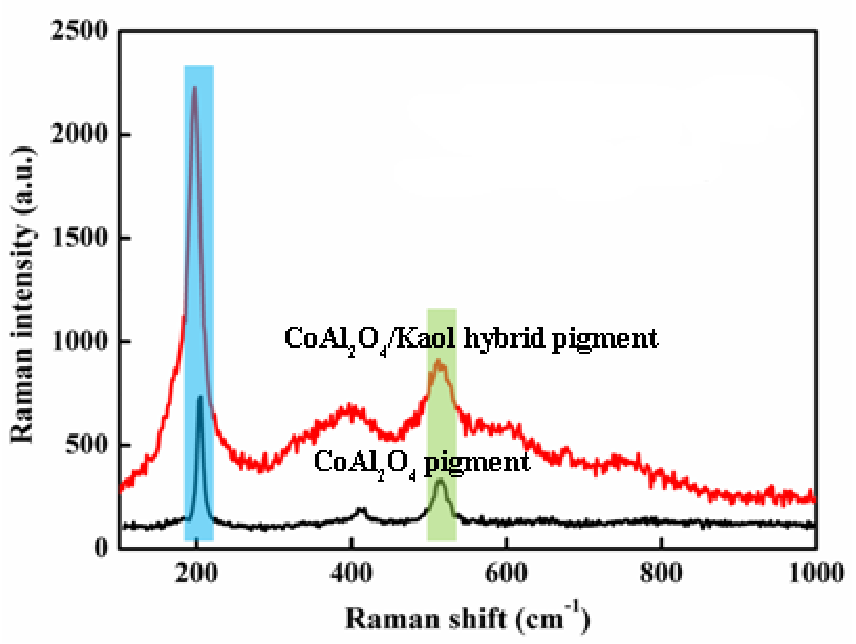

Figure S2. Raman spectra of $CoAl_2O_4$ pigment and $CoAl_2O_4$/Kaol hybrid pigment calcined at 1100 ℃.

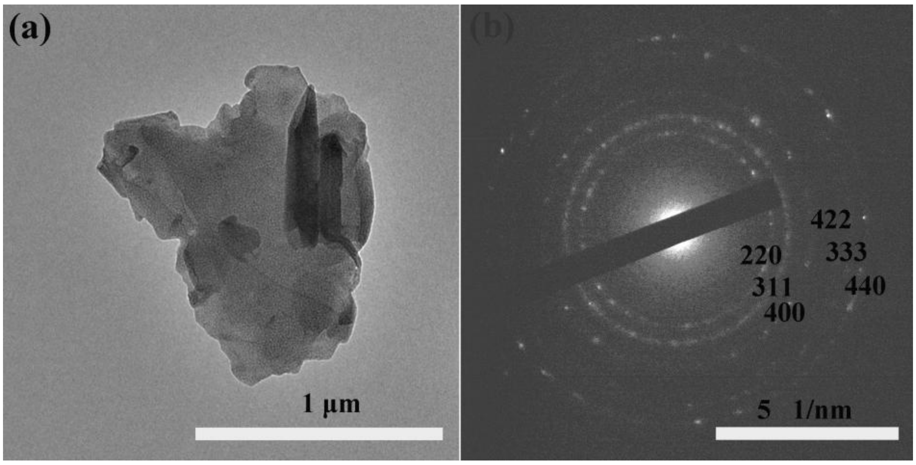

Figure S3. (a) TEM image of Kaol calcined at 1100°C and (b) the selected area electron diffraction pattern of hybrid pigment.

Table S2 CIE parameters of hybrid pigments prepared using 2 g Kaol and different contents of Co_3O_4 in the absence of Al_2O_3.

Number	Co_3O_4 (%)	CIE		
		L^*	a^*	b^*
a	0	92.6	0.581	4.456
b	2.4	64.502	-5.447	-29.504
c	4.8	54.919	-7.324	-41.085
d	9.1	41.680	-15.643	-40.693
e	13.0	36.303	-19.741	-41.774
f	16.7	1.856	-4.978	-0.174

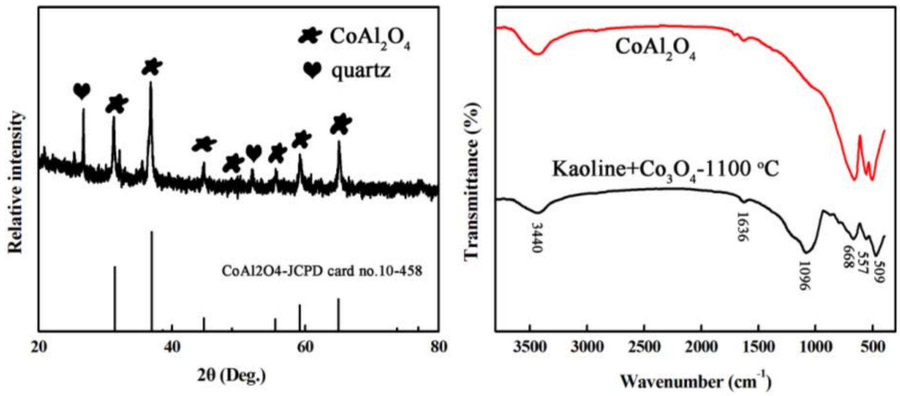

Figure S4. (a) XRD pattern and (b) FTIR spectra of $CoAl_2O_4$ and the as-prepared sample prepared using 13% Co_3O_4 and Kaol in the absence of Al_2O_3.

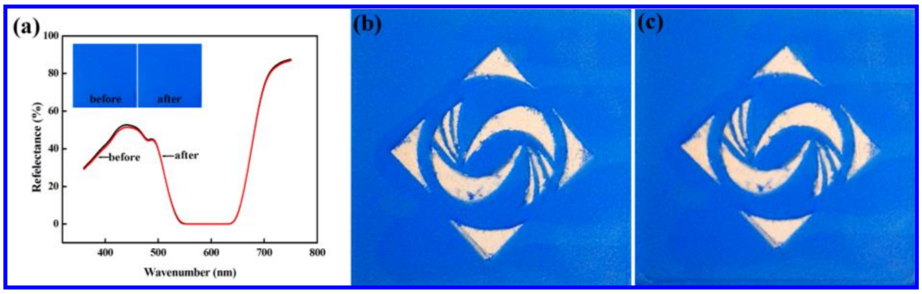

Figure S5. (a) UV-vis reflectance spectra of $CoAl_2O_4$/kaoline hybrid pigment before and after being exposed in UV accelerated weathering tester for 15 days (the insert is the photographs of hybrid pigment before and after being exposed under UV light). (b) and (c) Digital photos of $CoAl_2O_4$/kaoline hybrid pigment before and after being calcined at 1000 ℃.

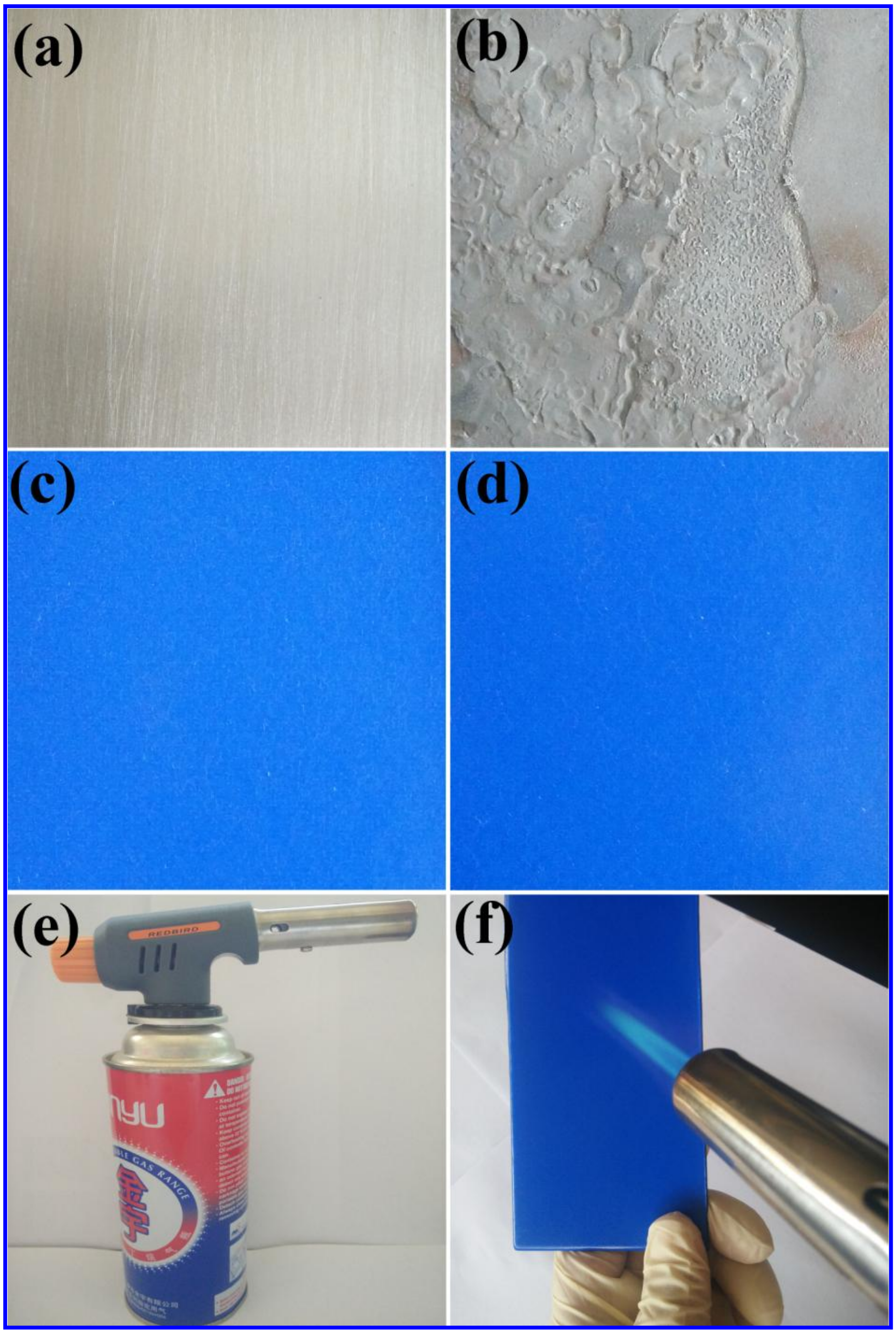

Figure S6. Digital photos of steel plates coated the commercial thermal resistance paint (a and b), and the one containing hybrid pigment (c and d) before and after being calcined at 800°C for 2 h. The image of (e) flame gun and (f) the commercial thermal resistance paint containing hybrid pigment after being heated, respectively.

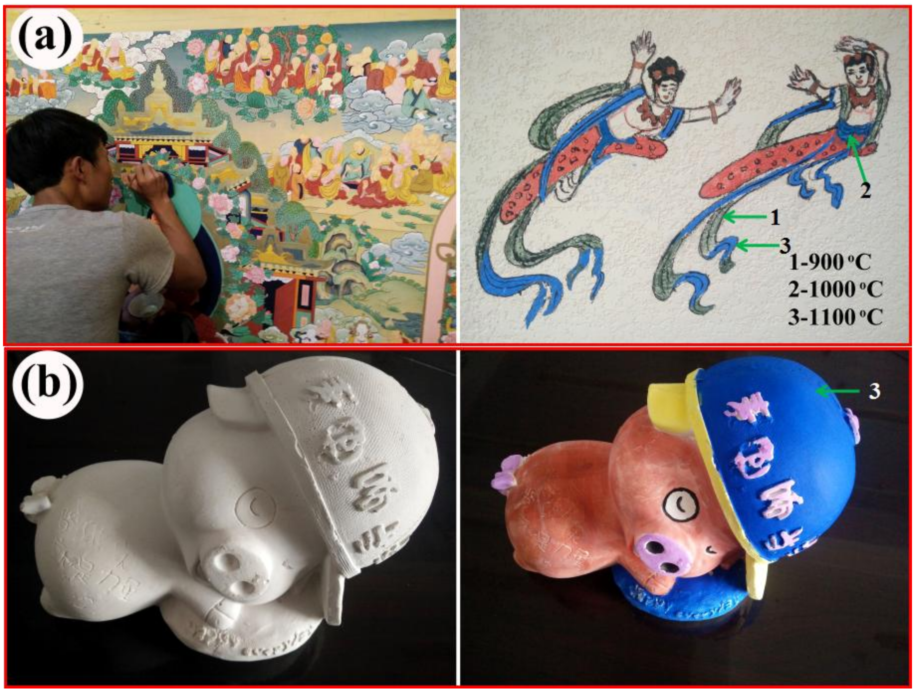

Figure S7. $CoAl_2O_4$/kaoline hybrid pigments prepared at different temperatures applied as a painting (a) and art pigment (b).

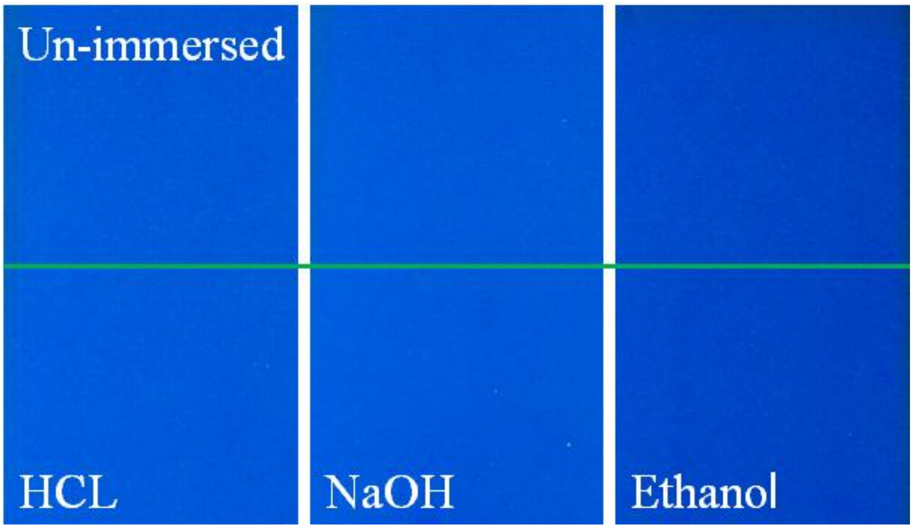

Figure S8. Digital photos of the sample plate immersed in 3 mol/L HCl, 3 mol/L H_2SO_4, 3 mol/L NaOH and ethanol after 72 h, respectively.

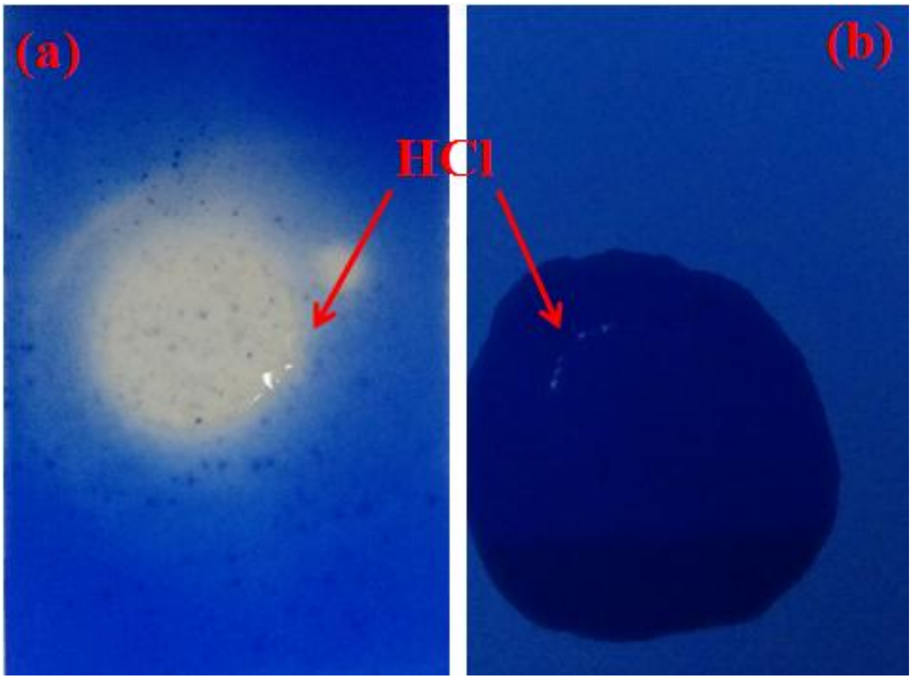

Figure S9. Digital photos of (a) ultramarine and (b) hybrid pigment after contacting with 36.5 wt% HCl.

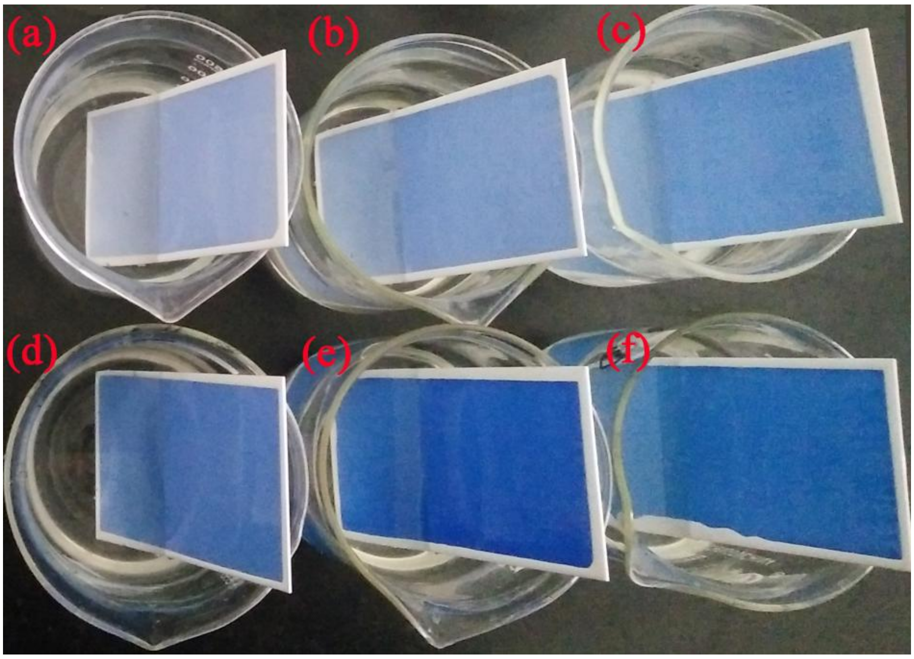

Figure S10. Digital photos of the anticorrosion coating containing (a) 1%, (b) 2%, (c) 3%, (d) 4%, (e) 5% hybrid pigment and (f) 5% $CoAl_2O_4$ pigment calcined at 1200^oC, respectively.

Table S3 Paint formulations

Materials	1	2	3	4	5
Epoxy Resin	60	60	60	60	60
Kaoline	5	-	-	-	-
Zno	-	5	-	-	-
Hybrid pigment	-	-	5	-	-
$CoAl_2O_4$-1100 ℃	-	-	-	5	-
Fe_2O_3	-	-	-	-	5
Additives	1	1	1	1	1
Fillings	15	15	15	15	15
Solvent	19	19	19	19	19
Total	100	100	100	100	100
